# Clinicopathological correlation of immune response in human cancers

**DOI:** 10.18632/oncotarget.27231

**Published:** 2019-10-08

**Authors:** Yuexin Liu

**Affiliations:** ^1^Department of Bioinformatics and Computational Biology, The University of Texas MD Anderson Cancer Center, Houston, Texas, USA

**Keywords:** clinicopathological characteristics, immune response, immunogenicity, cancer

## Abstract

**Background:**

The clinicopathologic association of tumor immune response is largely unknown. We systematically investigated this matter in human cancers.

**Results:**

Different cancer types exhibited distinct immune gene profiling. Four cancer types exhibited a significant and positive correlation of immune response with patient age. Significant but inconsistent correlation of immune response was observed with gender, surgical stage, and TNM stage in a small number of cancer types. In contrast, the histological grade appears to have much stronger and more consistent association with immune response as compared to the other clinicopathologic factors. Specifically, patients with high grade had significantly higher immune responses than those with low grade in 5 out of 12 analyzed cancer types. In addition, both histological and molecular classifications had a significant and strong association with tumor immune response.

**Methods:**

t-distributed stochastic neighbor embedding was used to assess similarity of immune gene profiling in human cancers. The Mann-Whitney or Kruskal-Wallis test was, respectively, used to compare the tumor immune response in two or more groups that were stratified by patient clinicopathological characteristics, such as gender, grade, stage (including surgical and TNM stage), histology, and molecular subtypes. Spearman correlation with student’s *t*-test was used to examine the association of patient age with immune response. Multiple tests with the Benjamini-Hochberg correction also were performed.

**Conclusions:**

Tumor grade should be taken into account in selection of patient candidates for immunotherapy. Prospective verification is needed before use of the findings for clinical practice.

## INTRODUCTION

Cancer immunotherapy is an attractive treatment strategy and associated with improved clinical outcomes in multiple cancer types [1, 2]. Immune checkpoint blockade therapy is promising in generating long-lasting responses in different cancer types [3, 4]. Biomarkers have been developed to predict patients’ responsivity to immunotherapy treatments, such as genome-wide mutational load [2], PD-L1 protein expression [5] in infiltrating immune cells, the number of cytotoxic T cells in a tumor microenvironment [6], or MSI status [7]. Currently, the clinicopathologic characteristics, such as tumor metastasis and surgical stage, are the main factors in selecting patients for immune checkpoint inhibitor therapy. For instance, ipilimumab (monoclonal antibody against CTLA-4 protein) was used for metastatic melanoma patients with unresectable stage III or stage IV disease [8]. Nivolumab (monoclonal antibody against PD-1 protein) is used for metastatic melanoma, metastatic squamous non-small cell lung cancer [9, 10], or head and neck cancer and bladder cancer with advanced disease. Atezolizumab (monoclonal antibody against the PD-L1 protein) is used for non-small cell lung cancer with metastasis [11], and the FDA recently approved it for treating triple-negative breast cancer with advanced disease [12]. Similarly, durvalumab (antibody against PD-L1 protein) is approved for treating patients with locally advanced or metastatic urothelial carcinoma [13]. Recently, our group found that activated immune response was significantly associated with high-grade disease in endometrial cancer [14]. Taken together, it appears that there is an intimate link between immune response and patients’ clinicopathological characteristics.

However, the clinicopathological association of immune response is largely unknown in many other cancer types, and it has not been systematically investigated in a wide array of human cancers. Moreover, many other clinicopathological features besides tumor stage or metastasis are not yet used as selection criteria for cancer immunotherapy. We recently devised an mRNA-based metric of preexisting immune conditions from the global immune gene signature [15] and systematically investigated the association of tumor immune response and patient outcome in human cancers [manuscript under review]. In this study, we build on this work and used the same patient cohort and the devised immune metrics to systematically examine the relationship between tumor immune response and patient clinicopathological characteristics, such as age, gender, histologic grade, and tumor stage (including surgical and TNM stage). The relationship between immune response and histological or molecular subtypes that have been used in clinical practice was also examined. Our results identify clinicopathological features that are strongly and significantly correlated with tumor immune response and have great potential as selection factors for cancer immunotherapy.

## RESULTS

### Dissimilarity of immune gene profiling across human cancers

**Figure 1:**
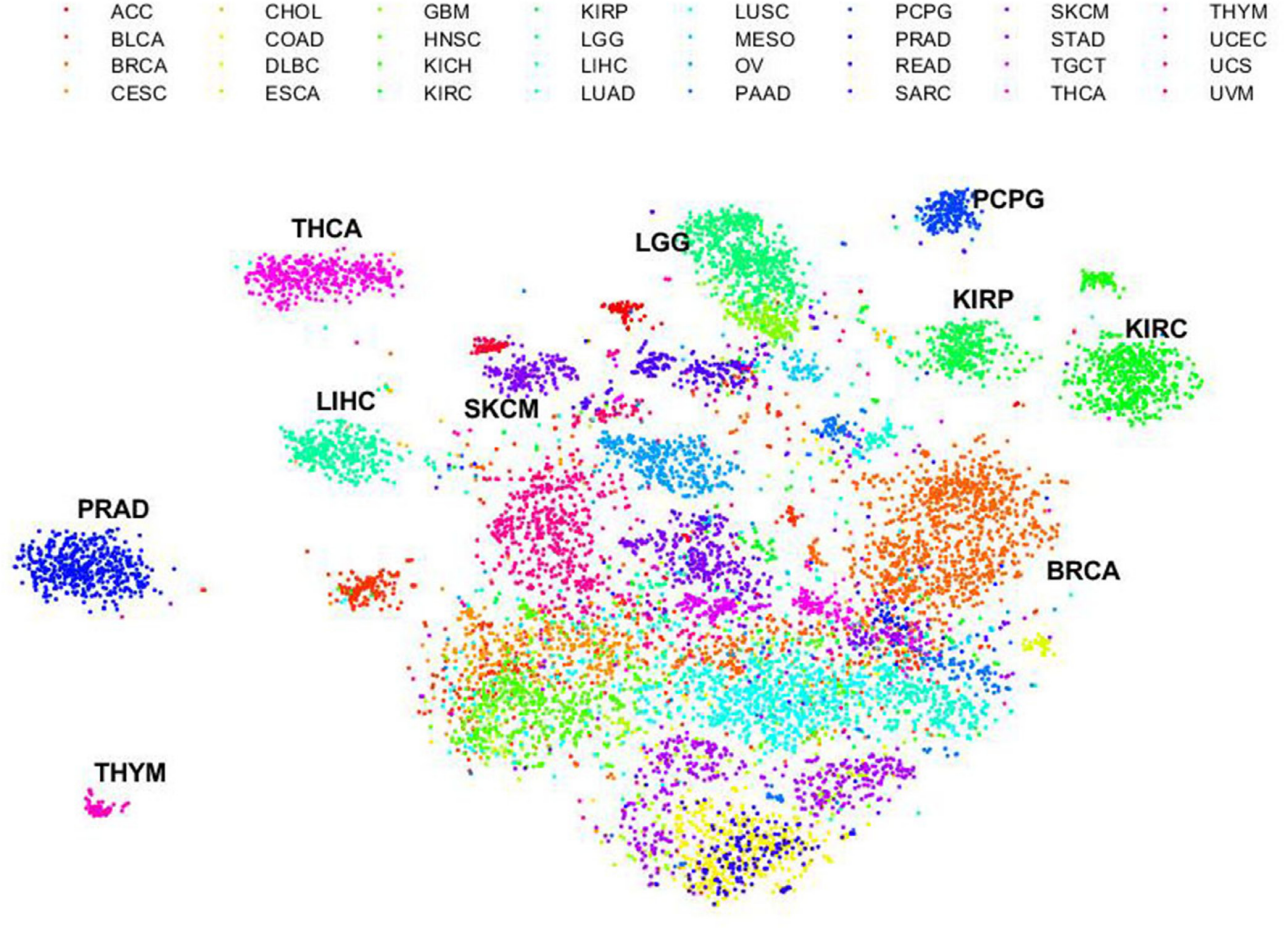
Dissimilarity of immune gene expression profiling across human cancers. Unsupervised t-SNE on expression profiling of the 382 immune signature genes across all cancer types. Each dot represents a sample. Color represents the cancer types shown on the right. ACC: Adrenocortical carcinoma; BLCA: bladder urothelial carcinoma; BRCA: breast invasive carcinoma; CESC: cervical squamous cell carcinoma and endocervical adenocarcinoma; CHOL: cholangiocarcinoma; COAD: colon adenocarcinoma; DLBC: lymphoid neoplasm diffuse large B-cell lymphoma; ESCA: esophageal carcinoma; GBM: glioblastoma multiforme; HNSC: head and neck squamous cell carcinoma; KICH: kidney chromophobe; KIRC: kidney renal clear cell carcinoma; KIRP: kidney renal papillary cell carcinoma; LGG: brain lower grade glioma; LIHC: liver hepatocellular carcinoma; LUAD: lung adenocarcinoma; LUSC: lung squamous cell carcinoma; MESO: mesothelioma; OV: ovarian serous cystadenocarcinoma; PAAD: pancreatic adenocarcinoma; PCPG: pheochromocytoma and paraganglioma; PRAD: prostate adenocarcinoma; READ: rectum adenocarcinoma; SARC: sarcoma; SKCM: skin cutaneous melanoma; STAD: stomach adenocarcinoma; TGCT: testicular germ cell tumors; THCA: thyroid carcinoma; THYM: thymoma; UCEC: uterine corpus endometrial carcinoma; UCS: uterine carcinosarcoma; UVM: uveal melanoma.

To assess the dissimilarity of immune gene expression profiling across human cancers, we carried out *t*-distributed stochastic neighbor embedding (t-SNE) on the 10,062 PanCanAtlas tumor samples and expression data of the 382 global immune signature genes [15] (Figure 1). t-SNE is an algorithm for dimensionality reduction by embedding high-dimensional points in low dimensions in a way that respects similarities between points [16]. As seen from the figure, samples from the same cancer type tend to group together and in addition cell origin provides the dominant signal in grouping of samples as well [i.e., kidney renal papillary cell carcinoma (KIRP) and kidney renal clear cell carcinoma (KIRC)]. Regardless, we found that some cancer types exhibited distinct immune gene expression profiling and were far apart from the others such as thymoma (THYM), prostate adenocarcinoma (PRAD), liver hepatocellular carcinoma (LIHC), thyroid carcinoma (THCA), skin cutaneous melanoma (SKCM), brain lower grade glioma (LGG), pheochromocytoma and paraganglioma (PCPG), KIRP, and KIRC. In addition, breast invasive carcinoma (BRCA) was widely separated from PRAD in the plot likely because of gender effect.

### Association of immune response with patient age

**Figure 2:**
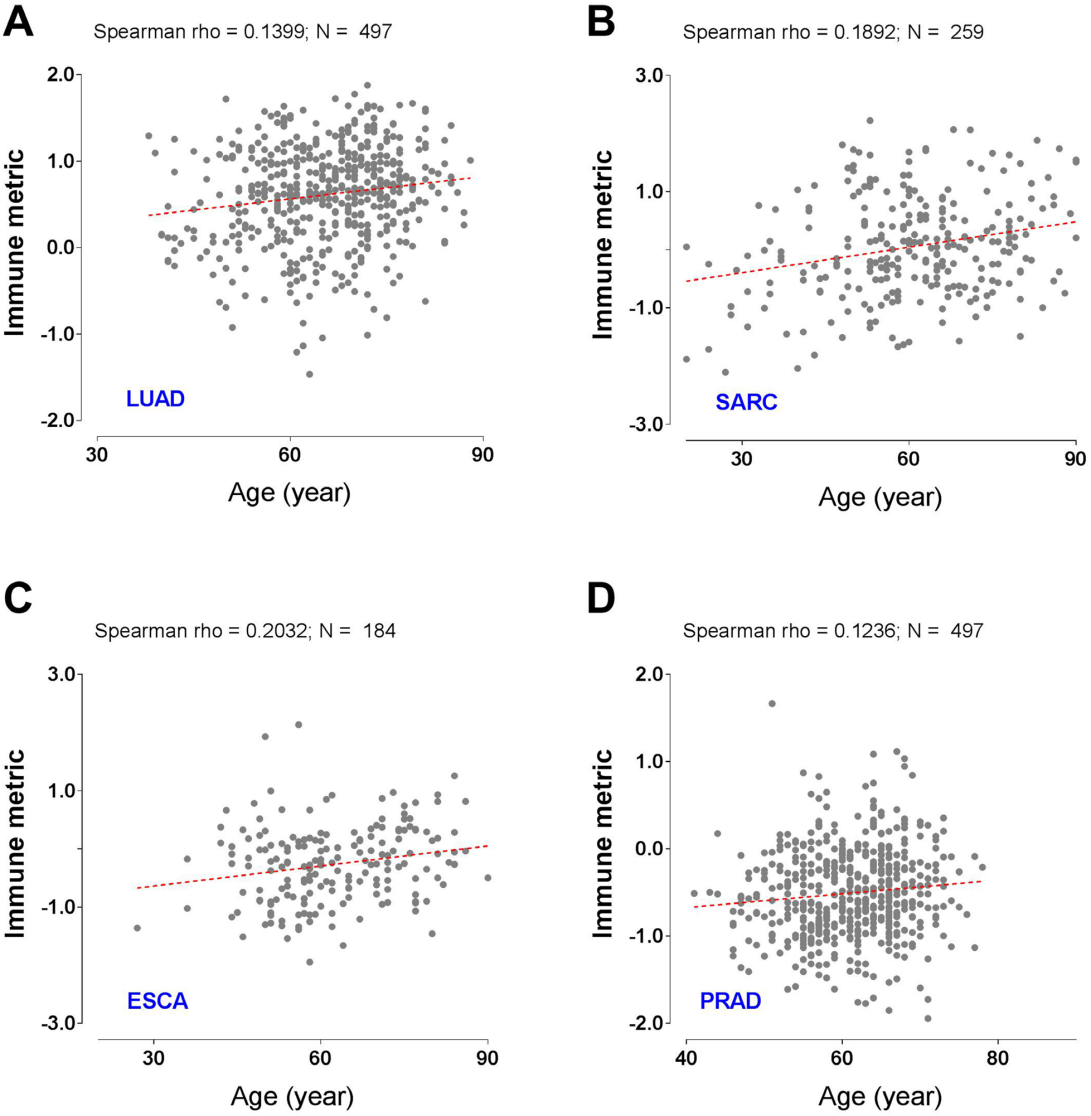
Tumor types with significant correlation of tumor immune response with patient age. Scatter plots with the best-fit lines for (**A**) lung adenocarcinoma (LUAD), (**B**) sarcoma (SARC), (**C**) esophageal carcinoma (ESCA), and (**D**) prostate adenocarcinoma (PRAD). N denotes the number of analyzed patients.

We generated a quantitative metric of tumor immune response by taking the median of gene expression values included in the global immune gene signature [15]. To examine the dependence of immune response upon patient age, we used the Spearman’s rank-order correlation and found that immune response was significantly correlated with patient age in an unadjusted model in eight cancer types. After the BH correction, the following four cancer types remain significant (*P* < 0.05, Student’s *t*-test, adjusted, Figure 2): lung adenocarcinoma (LUAD, *P* = 0.036, adjusted, Figure 2A), sarcoma (SARC, *P* = 0.036, adjusted, Figure 2B), esophageal carcinoma (ESCA, *P* = 0.046, adjusted, Figure 2C), and prostate adenocarcinoma (PRAD, *P* = 0.046, adjusted, Figure 2D). All of these correlations were positive, meaning that older patients had a significantly larger immune response than younger ones. However, the overall impact of patient age on immune response is not strong, indicated by the small correlation coefficients. Of note, the statistical significance also should be interpreted with caution, as it is strongly dependent on the number of analyzed samples.

### Association of immune response with gender

**Figure 3:**
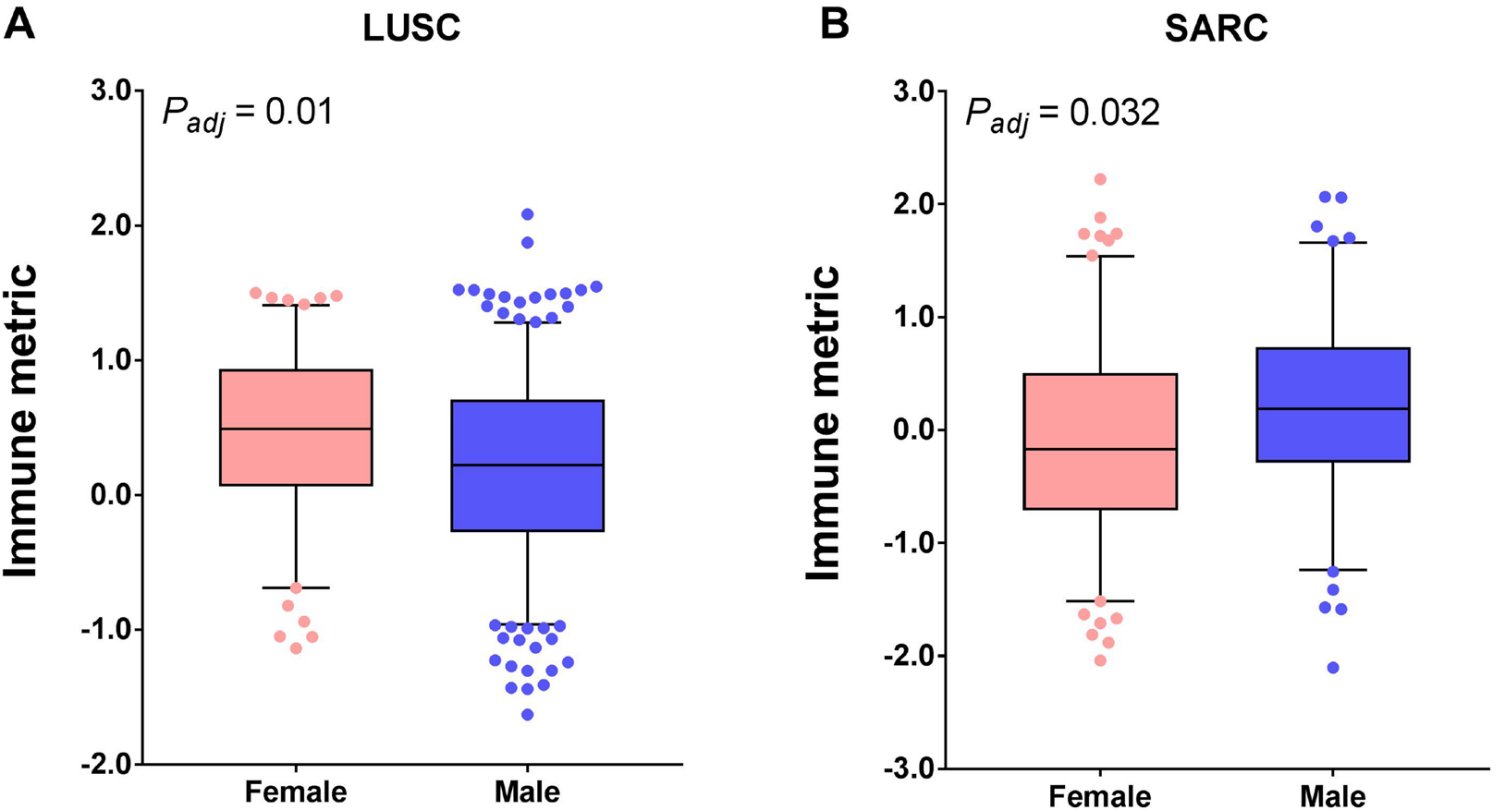
Tumor types with significant correlation of tumor immune response with gender. Boxplots for (**A**) lung squamous cell carcinoma (LUSC) and (**B**) sarcoma (SARC). The central line of each box is the median value, and the edges are the 25th and 75th percentiles. The whiskers extend to the 5th and 95th percentiles, and data points outside the whiskers are plotted individually as dots.

To examine the dependence of tumor immune response on patient gender, next we used the Mann-Whitney test to compare the immune response between female and male patients. We found that a total of nine cancer types exhibited statistically a significant difference in an unadjusted model. After multiple testing corrections, two cancer types remained significant (*P* < 0.05, Mann-Whitney test, adjusted, Figure 3). In particular, immune response was greater in women than in men for lung squamous cell carcinoma (LUSC), while sarcoma (SARC) showed the opposite trend.

### Association of immune response with histological grade

**Figure 4:**
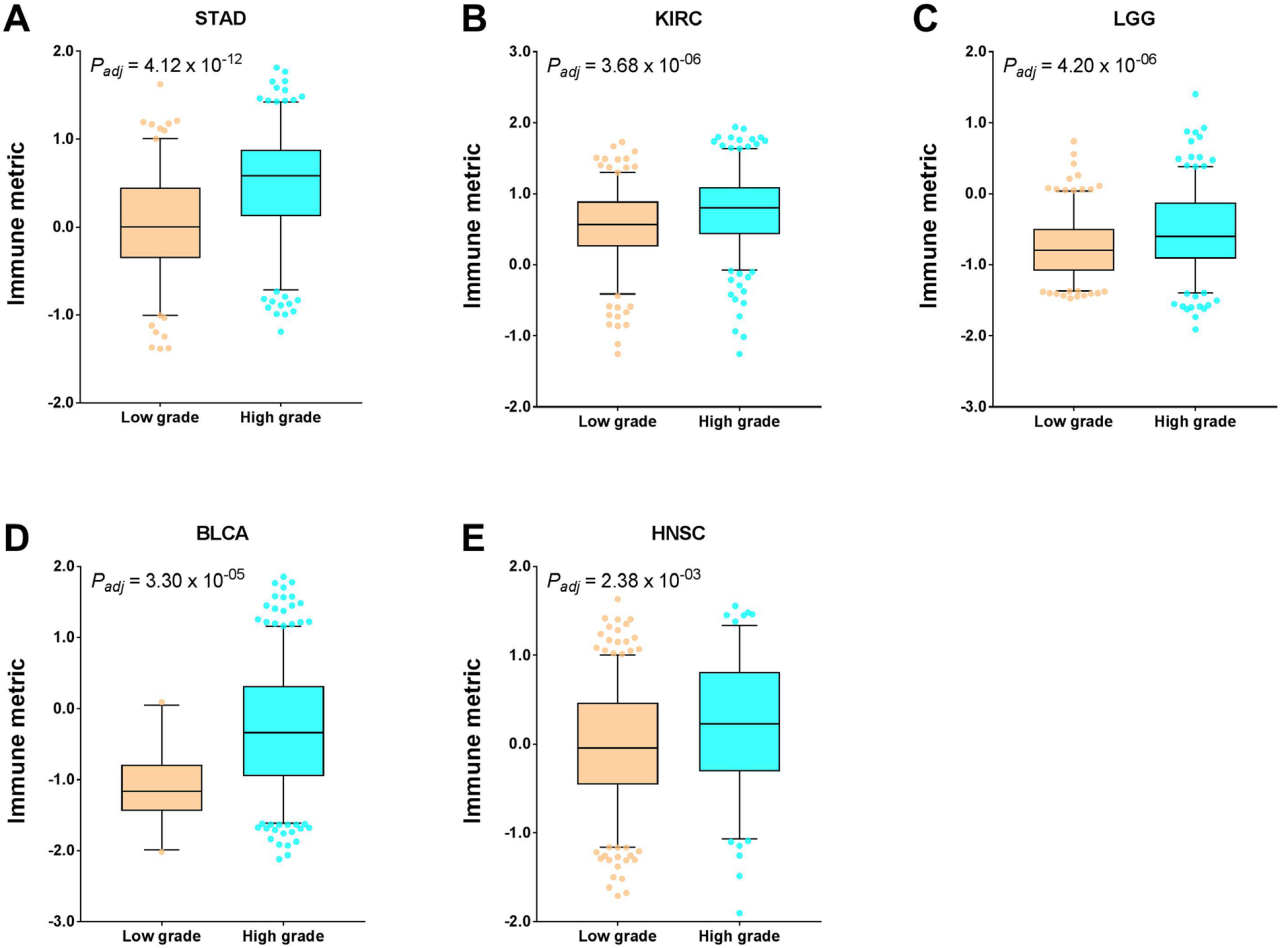
Tumor types with significant correlation of tumor immune response with histological grade. Boxplots for (**A**) stomach adenocarcinoma (STAD), (**B**) kidney renal clear cell carcinoma (KIRC), (**C**) brain lower grade glioma (LGG), (**D**) bladder urothelial carcinoma (BLCA), and (**E**) head and neck squamous cell carcinoma (HNSC). The central line of each box is the median value, and the edges are the 25th and 75th percentiles. The whiskers extend to the 5th and 95th percentiles, and data points outside the whiskers are plotted individually as dots.

Using a similar method as described above, next we examine the association of histological grade with tumor immune response. For some reason, only 12 cancer types had the histological grade information, but half of them exhibited a significant immune response dependence on the tumor grade in an unadjusted model. After multiple testing corrections, five cancer types remained significant. Prominently, the immune response was consistently higher in patients with a high-grade disease than in those with a low-grade disease in all five of the following cancer types: stomach adenocarcinoma (STAD, *P* = 4.12 × 10^-12^, adjusted); kidney renal clear cell carcinoma (KIRC, *P* = 3.68 × 10^-06^, adjusted); brain lower grade glioma (LGG, *P* = 4.20 × 10^-06^, adjusted); bladder urothelial carcinoma (BLCA, *P* = 3.30 × 10^-05^, adjusted); and head and neck squamous cell carcinoma (HNSC, *P* = 2.38 × 10^-05^, adjusted) (Figure 4). Moreover, the small *P* values and large median differences in immune response between high- and low-grade patients indicated a dramatic influence of the histological grade on tumor immune response, and patients with high grade may be ideal candidates for immunotherapy.

### Association of immune response with tumor stage

**Figure 5:**
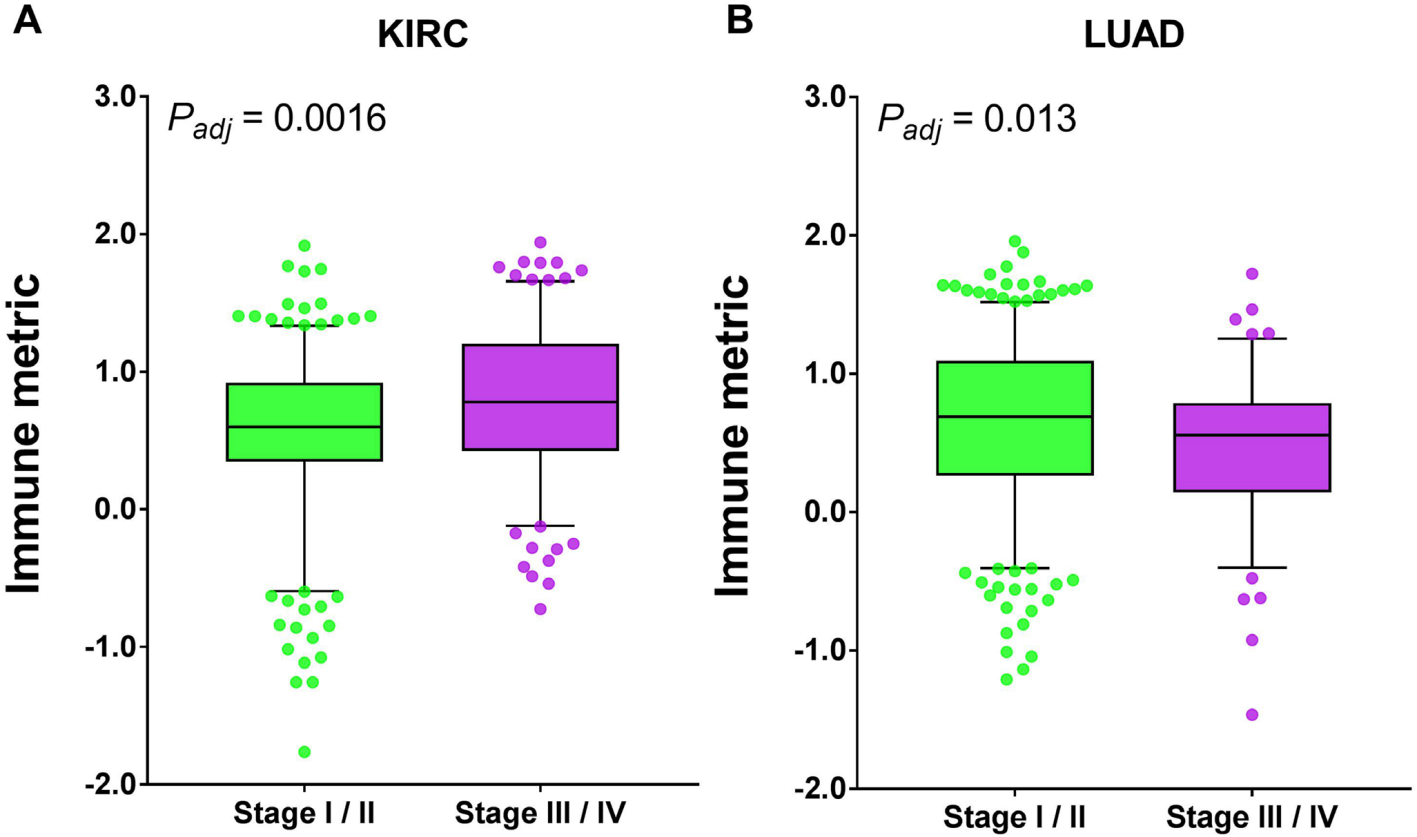
Tumor types with significant correlation of tumor immune response with surgical stage. Boxplots for (**A**) kidney renal clear cell carcinoma (KIRC) and (**B**) lung adenocarcinoma (LUAD). The central line of each box is the median value, and the edges are the 25th and 75th percentiles. The whiskers extend to the 5th and 95th percentiles, and data points outside the whiskers are plotted individually as dots.

We first examined the association of tumor surgical stage with tumor immune response. Six and two out of 21 cancer types (with stage information) exhibited significant correlation of immune response with surgical stage before and after multiple testing corrections, respectively. Different from histological grade, the association of surgical stage with immune response is not consistent among human cancers. In particular, kidney renal clear cell carcinoma (KIRC) patients with advanced-stage disease had significantly higher immune response than those with early-stage disease (*P* = 0.0016, adjusted), while lung adenocarcinoma (LUAD) patients showed the opposite trend (*P* = 0.013, adjusted) (Figure 5). Compared with histological grade, surgical stage had a limited impact on tumor immune response, as evidenced by the *P* values and median immune differences.

**Figure 6:**
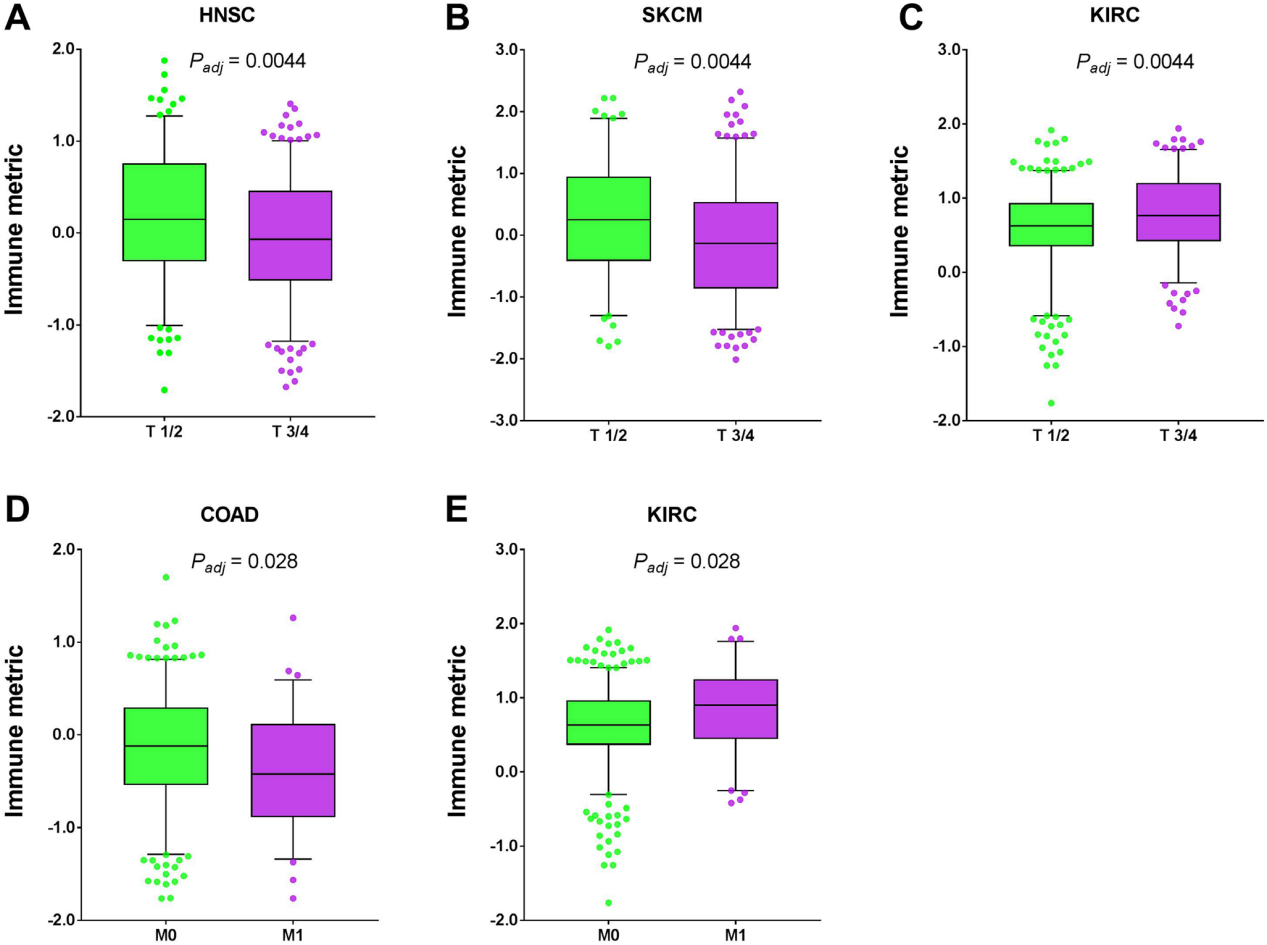
Tumor types with significant correlation of tumor immune response with TNM stage. Boxplots for (**A**) head and neck squamous cell carcinoma (HNSC), (**B**) skin cutaneous melanoma (SKCM), (**C**) kidney renal clear cell carcinoma (KIRC), (**D**) colon adenocarcinoma (COAD), and (**E**) kidney renal clear cell carcinoma (KIRC). The central line of each box is the median value, and the edges are the 25th and 75th percentiles. The whiskers extend to the 5th and 95th percentiles, and data points outside the whiskers are plotted individually as dots.

Next we examined the relationship between TNM stage and tumor immune response. After multiple testing correction, we found that three cancer types exhibited significant correlation of immune response with tumor T stage, two cancer types with tumor M stage, and none with tumor N stage (Figure 6). Similar to surgical stage, the impact of TNM stage on immune response is not consistent either. In head and neck squamous cell carcinoma (HNSC, *P* = 0.0044, adjusted) and skin cutaneous melanoma (SKCM, *P* = 0.0044, adjusted), patients with T3/4 had significantly lower immune response than those with T1/2, while kidney renal clear cell carcinoma (KIRC, *P* = 0.0044, adjusted) showed the opposite trend. The colon adenocarcinoma (COAD, *P* = 0.028, adjusted) patients with M1 had significantly lower immune response than those with M0, while kidney renal clear cell carcinoma (KIRC, *P* = 0.028, adjusted) showed the opposite trend.

### Association of immune response with histological or molecular subtype

**Figure 7:**
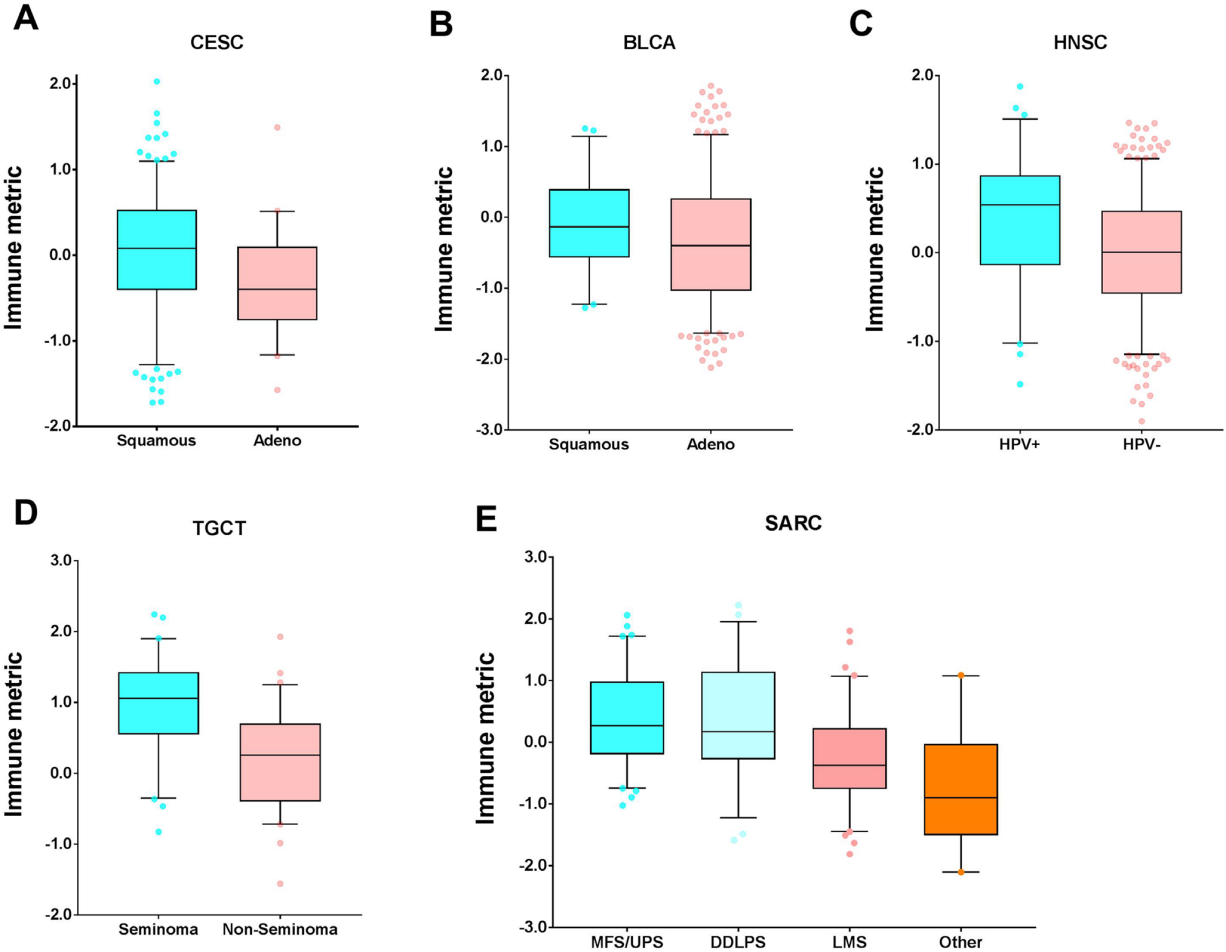
Tumor types with significant correlation of tumor immune response with histology. Boxplots for (**A**) cervical squamous cell carcinoma and endocervical adenocarcinoma (CESC), (**B**) bladder urothelial carcinoma (BLCA), (**C**) head and neck squamous cell carcinoma (HNSC), (**D**) testicular germ cell tumor (TGCT), and (**E**) sarcoma (SARC). The central line of each box is the median value, and the edges are the 25th and 75th percentiles. The whiskers extend to the 5th and 95th percentiles, and data points outside the whiskers are plotted individually as dots.

Next we examined the relationship between tumor immune response and histological or molecular classifications in human cancers that have been used in clinical practice [17]. Approximately 15.5% of cervical squamous cell carcinoma and endocervical adenocarcinoma (CESC) cases were adenocarcinoma, which had significantly lower immune responses than those with squamous histology (*P* = 4.09 × 10^-05^, Mann-Whitney test, Figure 7A). The trend was consistent in bladder urothelial carcinoma (BLCA), though not statistically significant (*P* = 0.09, Figure 7B). In head and neck squamous cell carcinoma (HNSC), approximately 15.0% were HPV+, which had a significantly higher immune response than the HPV- cases (*P* = 1.02 × 10^-05^, Figure 7C). In testicular germ cell tumor (TGCT), about 48.0% were seminoma and the others were non-seminoma. Compared with the non-seminoma cases, the seminoma patients had a significantly higher immune response (*P* = 3.33 × 10^-09^, Figure 7D). Consistently, it was recently reported that extensive immune infiltration was noted in the seminoma samples by the TCGA effort [18]. After excluding those with no histology information, we obtained 229 sarcoma (SARC) that had immune response data, among which 46 (~20.1%) were dedifferentiated liposarcoma (DDLPS), 83 (~36.2%) were leiomyosarcoma (LMS), 80 (~34.9%) were myxofibrosarcoma/ undifferentiated pleomorphic sarcoma (MFS/UPS), and 20 (~8.7%) had other histology. Interestingly, there were significant differences in immune response among these four histological subtypes (*P* = 3.84 × 10^-09^, Kruskal Wallis test, Figure 7E), with UPS/MFS and DDPLS subtypes having the highest immune response. This observation is in agreement with the previous report [19]. The uterine corpus endometrial carcinoma (UCEC) patients were mainly categorized into two histological subtypes, serous versus endometrioid. However, these two histologic subtypes did not show a difference in immune response (data not shown).

**Figure 8:**
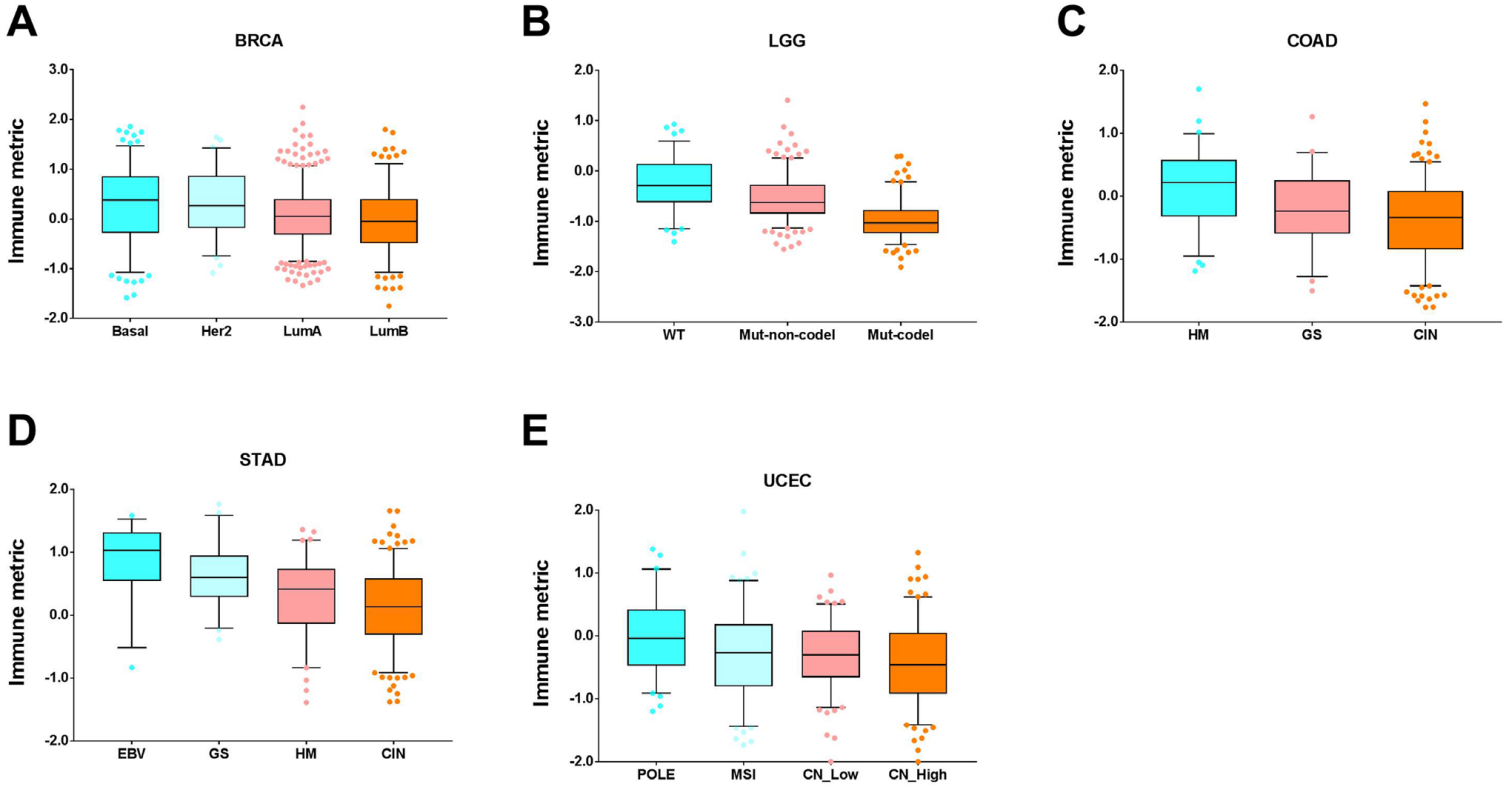
Tumor types with significant correlation of tumor immune response with molecular subtypes. Boxplots for (**A**) breast invasive carcinoma (BRCA), (**B**) brain lower grade glioma (LGG), (**C**) colon adenocarcinoma (COAD), (**D**) stomach adenocarcinoma (STAD), and (**E**) uterine corpus endometrial carcinoma (UCEC). The central line of each box is the median value, and the edges are the 25th and 75th percentiles. The whiskers extend to the 5th and 95th percentiles, and data points outside the whiskers are plotted individually as dots.

We next correlated tumor immune response with well-established molecular subtypes and found a significant association in several cancer types. The breast invasive carcinoma (BRCA) patients were mainly categorized into four subtypes (Basal, Her2, LumA, and LumB) following the PAM50 classification, which exhibited a statistically significant difference in tumor immune response (*P* = 7.59 × 10^-07^, Kruskal-Wallis test, Figure 8A). It appears that the Basal group had a significantly higher immune response than either the LumA or LumB groups. The Basal cases had comparable immune responses with the Her2 cases. In brain lower grade glioma (LGG), the IDH wide-type (wt) cases had the significantly highest immune response, followed by the IDHmut-non-codel cases (*P* = 1.52 × 10^-31^, Figure 8B). It appears that the hypermutated (HM) colon adenocarcinoma (COAD) patients had the significantly highest immune response among the three different molecular subtypes (CIN, GS, HM) (*P* = 1.04 × 10^-06^, Figure 8C). In addition to the three subtypes in colon adenocarcinoma (COAD), stomach adenocarcinoma (STAD) had an additional subtype, named EBV. Similar to COAD, the CIN cases had the lowest immune response. Different from COAD, the GS case had a significantly higher immune response than the HM cases in stomach adenocarcinoma (STAD). Also, the EBV had the highest immune response (*P* = 2.01 × 10^-11^, Figure 8D). There were statistically significant differences in immune response among these four uterine corpus endometrial carcinoma (UCEC) subtypes (*P* = 0.0001, Figure 8E), in which the POLE (DNA polymerase epsilon) cases had the highest immune response and this observation is consistent with a previous report [20].

## DISCUSSION

In this study, we have assessed the dissimilarity of immune gene profiling in human cancers and applied the immune response devised in our previous work to systematically investigate its dependence upon patient clinicopathological characteristics. We found that associations of immune response with age, gender, surgical stage, and TNM stage are relatively weak, and most times inconsistent. In contrast, grade, histology, and molecular classifications have a consistent and much more dramatic impact on tumor immune response. Our results provide a comprehensive view of the relationship between immune response and clinicopathological features, and are helpful in clinical decision-making regarding immunotherapy treatment.

Although tumor stage is one of the factors for choosing patients for immunotherapy, our results showed that it was significantly associated with immune response in only 2 out of 21 examined cancer types. Moreover, the associative patterns were not consistent among cancer types. We found that tumor grade, however, was significantly associated with immune response in 5 out of 12 examined cancer types. Moreover, these correlations were all positive and relatively strong. Therefore, it is important for patients’ histological grade to factor into the immunotherapy option decision. However, only 12 cancer types had the histological grade data available.

Chemotherapy remains the main treatment for triple-negative breast cancer (TNBC), and no targeted therapies are available, due to the molecular characteristics of this disease. Our results showed that the breast cancer basal group (highly concordant with TNBC) had significantly higher immune response than LumA/B breast cancer patients, suggesting that these patients (basal or TNBC) might be good candidates for currently emerging immune checkpoint inhibitor therapy. Consistent with this finding, it was recently reported that atezolizumab (antibody against PD-L1 protein) was shown to prolong progression-free survival; the drug was recently approved by FDA for treatment of metastatic TNBC patients [12].

Our results further demonstrated that the hypermutated (HM) subtype in colon adenocarcinoma (COAD) had a significantly higher immune response than either the chromosomal instability (CIN) or genome stable (GS) subtypes, making such patients potential candidates for immunotherapeutic treatments. Given that the majority of these hypermutated (HM) cases were MSI-high (MHS-H) tumors, these data were well consistent with previous reports that the colon adenocarcinoma (COAD) cases with MSI-H were typically excellent candidates for immunotherapy [7]. In addition, the POLE group has the highest immune response among the four molecular subtypes in uterine corpus endometrial carcinoma (UCEC), suggesting that patients in this group may be good candidates for immunotherapy. This also is consistent with a previous report that the EC patients with POLE mutation elicited anti-tumor immune response [20]. The EVB cases in stomach adenocarcinoma (STAD) and the IDH wide-type cases in brain lower grade glioma (LGG) had the highest immune response, suggesting that these patients are likely to be good candidates for immunotherapy.

Our results showed that liver hepatocellular carcinoma (LIHC) had distinct immune expression profiling [21] but there was no significant correlation of immune response with either patient gender or grade. Furthermore, we found that LIHC patients with stage I/II disease had higher immune response than those with stage III/IV disease, but with marginally statistical significance (unadjusted *P* = 0.07), suggesting that early-stage LIHC patients were likely favorable candidates for currently emerging immunotherapy [22]. In contrast, there are no optimal treatments for advanced-stage LIHC patients [23] who need novel therapeutic strategies [24]. Accumulating evidences showed that metabolic dysreuglaiton plays an important role not only in the LIHC tumorigenesis [25] but also in the presence of certain lymphocyte populations [26]. It’s therefore speculated that reprogramming the metabolic qualities of anti-tumor immune cells might be an alternate approach to improve immunotherapy for the stage III/IV LIHC patients [27].

The present study has several limitations. First, it was a retrospective study. Second no patients in this study received immunotherapy, for which we cannot evaluate the clinical benefit of immune response in terms of responsivity. Still, these analyses provide useful insights into the relationship between clinicopathologic characteristics and tumor immune response and can guide further clinical trial development.

## MATERIALS AND METHODS

### Patient samples and immune metric

A total of 10,062 PanCanAtlas tumor samples covering 32 different cancer types were used in this study, with more details as described in our previous publication [15]. The corresponding immune metrics quantifying tumor immune response were from our recent work [under review]. The patients’ clinicopathological characteristics, as well as histological or molecular classifications, were obtained from the TCGA PanCanAtlas recent publications [17, 28].

### t-Distributed Stochastic Neighbor Embedding

To visualize the grouping of cancer samples, we performed t-Distributed Stochastic Neighbor Embedding (t-SNE) on the 10,062 TCGA PanCanAtlas tumor samples and the 382 immune genes we recently identified [15]. The expression data of these immune genes were first median centered and then log 2 transformed. We used “Barnes-Hut” optimization algorithm and the Correlation metric to generating the two-dimensional embedding data points. Other parameters included: perplexity = 100, MaxIter = 10,000, TolFun = 1e-10, Exaggeration = 20, LearnRate = 500.

### Correlation analysis of clinicopathological features

In this study, patient age was treated as a continuous variable and Spearman rank-order correlation was used to examine the relationship between tumor immune response and age. The difference in immune response between male and female patients was examined by using the Mann-Whitney test to assess the association of tumor immune response with gender.

To examine the relationship between tumor immune response and histological grade, we grouped patient samples annotated as either “G1”, “G2”, or “Low Grade” in the clinical file, categorized as “Low Grade.” Those annotated as either “G3”, “G4”, or “High Grade” are categorized as “High Grade.” All the other samples were excluded from the analysis. As a result, only 12 tumor types remained for the grade-immune response correlation analysis. Then the median difference in immune response between low-grade and high-grade group was compared.

Similarly, to correlate tumor immune response with surgical stage, we grouped patients samples annotated as either “Stage I”, “Stage IA”, “Stage IB”, “Stage II”, “Stage IIA”, “Stage IIB”, or “Stage IIC” in the clinical file, categorized as “Early Stage”. Those annotated as “Stage III” or “Stage IV” were categorized as “Advanced Stage”. All the other samples were excluded from the analysis. As a result, a total of 21 cancer types had surgical stage information. The median difference in immune response between the Early-Stage and Advanced-Stage group was compared.

To correlate tumor immune response with TNM stage, we first combined T1 and T2 together as the T1/2 group, and T3 and T4 together as the T3/4 group. Then the median difference in immune response between the T1/2 and T3/4 groups was compared. Similarly, we combined N0 and N1 together as the N0/1 group, and N2 and N3 together as the N2/3 group. Then the median difference in immune response between the N0/1 and N2/3 groups was compared. Finally, we compared the median difference in immune response between M0 and M1 groups.

### Correlation analysis of histological or molecular subtypes

In this study, we only chose those histological or molecular subtypes that have already been used in clinical practice [17] for the downstream analysis. The well-established histological subtypes included human papillomavirus (HPV) status in head and neck squamous cell carcinoma (HNSC) patients, squamous versus adenocarcinoma in cervical squamous cell carcinoma and endocervical adenocarcinoma (CESC) and bladder urothelial carcinoma (BLCA) patients, serous versus endometrioid in uterine corpus endometrial carcinoma (UCEC) patients, DDLPS versus LMS versus MFS/UPS in sarcoma (SARC) patients, and seminoma versus non-seminoma in testicular term cell tumor (TGCT) patients. For the same reason, the molecular subtypes investigated in detail in this study included: Basal/Her2/LumA/LumB in breast invasive carcinoma (BRCA) patients, CIN/GS/HM-indel/HM-SNV or /EBV in gastrointestinal cancer patients, IDH mutation status in glioma patients, and POLE/MSI/CN_Low/CN_High in uterine corpus endometrial carcinoma (UCEC) patients. If a subtype had a small number of patients, it was either excluded from the comparison analysis or combined with the other closely-related subtypes. For instance, the HM-SNV subtype in colon adenocarcinoma (COAD) had a small number of patients and was therefore combined with HM-indel to the HM subtype before comparison analysis. The Normal subtype in breast invasive carcinoma (BRCA) and IDH mutant group in glioblastoma multiforme (GBM) typically had a small number of patients, and as such were excluded from downstream analyses. We then used either the Mann-Whitney test or the Kruskal-Wallis test to compare the median difference in immune response among the histological or molecular subtypes, depending on the number of analyzed subtypes. The patients with no subtype information were excluded from the comparison analysis and percentage calculation.

### Statistical analysis

The Mann-Whitney test was used to assess statistical significance in immune response between the dichotic groups stratified by gender, grade, surgical stage, TNM stage, and histology (e.g., HPV status, squamous versus adeno, seminoma versus non-seminoma, serous versus endometrioid). The Kruskal-Wallis test was used to assess statistical significance in immune response among more than two groups, such as the PAM50 subtype in BRCA, molecular subtypes in gastrointestinal cancer and uterine corpus endometrial carcinoma (UCEC). The Student’s *t*-test was used to assess the statistical significance in the Spearman correlation. In all cases, multiple testing was performed with Benjamini-Hochberg (BH) correction. All statistical tests were two-sided, and a *P* value of less than 0.05 was considered significant. The calculations and graphs were made with GraphPad Prism, version 7.03 (GraphPad Software, Inc., La Jolla, CA).

## Abbreviations

CIN: Chromosomal instability
GS: genome stable
HM: hypermutated
MSI: microsatellite instability
EBV: Epstein-Barr virus
SNV: single-nucleotide variant
HPV: human papillomavirus
